# Effects of Coffee Extracts with Different Roasting Degrees on Antioxidant and Anti-Inflammatory Systems in Mice

**DOI:** 10.3390/nu10030363

**Published:** 2018-03-16

**Authors:** Sukyoung Choi, Soohan Jung, Kwang Suk Ko

**Affiliations:** 1 Department of Converging Clinical & Public Health, Ewha Womans University, Seoul 03760, Korea; chl931101@naver.com; 2 Department of Nutritional Science and Food Management, Ewha Womans University, Seoul 03760, Korea; tngks1231@gmail.com

**Keywords:** coffee extracts, roasting degree, anti-inflammatory response

## Abstract

Coffee roasting affects the taste, color, and aroma of coffee. The Maillard reaction, a major reaction during the roasting process, produces melanoidin, which affects the overall antioxidant capacity and anti-inflammatory effects of coffee. In this experiment, coffee roasting was divided into four degrees: Light, Medium, City, and French. To examine the in vivo antioxidant and anti-inflammatory effects of coffee extracts with different roasting degrees, we used 10-week-old male C57BL/6 mice. Mice were pre-treated with coffee extracts for 10 days by oral gavage (300 mg/Kg.B.W). After the last pre-treatment, lipopolysaccharide (LPS, 15 mg/Kg.B.W) was injected intraperitoneally for immune stimulation. Histopathological analysis showed that hepatic portal vein invasion and liver necrosis were severe in the LPS-treated group. However, these phenomena were greatly ameliorated when mice were pre-treated with Light- or Medium-roasted coffee extracts. Hepatic glutathione level was increased in the French group but decreased in the LPS-stimulated group. When mice were treated with LPS, mRNA expression level of tumor necrosis factor-alpha (TNF-α) was increased, whereas TNF-α expression was significantly reduced in the Light and Medium groups. Treatment with coffee extracts decreased the mRNA expression levels of interleukin 6 (IL-6) in mice stimulated by LPS, regardless of coffee roasting degrees. These effects decreased with the increasing coffee roasting degree. Results of luciferase reporter assay revealed that these effects of coffee extracts were transcriptionally regulated by the NF-κB pathway. Taken together, these results suggest that the roasting degree affects the antioxidant and anti-inflammatory effects of coffee extracts.

## 1. Introduction

Coffee, one of the most widely consumed beverages in the world, is a major dietary source of many bioactive substances [1]. Interestingly, many recent studies have shown that coffee consumption has potentially inverse correlations with chronic diseases such as cancer, cardiovascular disease, obesity, and diabetes [2,3,4,5]. Coffee contains diverse phenolic and non-phenolic compounds such as caffeine, chlorogenic acid, trigonelline, kahweol, and cafestol. These constituents of coffee are known to have similar effects to antioxidants in animals, which might be a reason for the disease-prevention benefit of coffee [6,7,8,9].

Roasting affects the taste, color, and aroma of coffee. During coffee roasting, components of coffee beans undergo chemical changes that ultimately affect the physiological effects of coffee. Recent reports have suggested that the proportion of chlorogenic acid, unlike caffeine, decreases as the roasting process continues [10,11]. At the same time, it has been demonstrated that the Maillard reaction, a major reaction occurring during the roasting process, produces melanoidins through the interaction of reducing sugars and free amino compounds in coffee beans. These newly-produced compounds such as hydroxyphenylindans, hydroxyl-dihydrocaffeic acid, and cinnamoyl-shikimic acids, including melanoidins, will affect the overall antioxidant capacity and anti-inflammatory effect of coffee [12,13,14,15].

It has become increasingly evident that chronic diseases are closely related to the cellular damage caused by excessive oxidative stress. Reactive oxygen species (ROS), including hydroxyl radical products such as superoxide anion radical (*O_2_⁻), and hydroxyl radical (OH⁻*) generated by intracellular aerobic metabolism and environmental stimuli, are the major cause of oxidative damage and severe inflammation in animals [16,17]. Activated inflammatory responses stimulate the signaling of nuclear factor-kappa B (NF-κB), one of the key transcription factors of inflammation, which initiates inflammatory response via increasing expression of pro-inflammatory cytokines such as tumor necrosis factor-alpha (TNF-α), interleukin 6 (IL-6), interleukin 1-beta (IL-1β), and inducible nitric oxide synthase (iNOS) [18,19]. As a defense system against inflammation, regulation of expression of cytoprotective genes is mediated by the nuclear factor erythroid 2-related factor 2 (Nrf2)-antioxidant response element (ARE) transcription regulation system to produce internal antioxidant substances such as glutathione synthesizing enzymes, glutathione transferases (GSTs), NAD(P)H: quinone oxidoreductase 1 (NQO1), and heme oxygenase 1 (HO-1) [20].

Several studies have reported that the antioxidant capacity of coffee is especially influenced by caffeine and chlorogenic acid [21,22]. It has also been suggested that coffee can reduce the expression of pro-inflammatory cytokines in vivo with a protective effect against lipid peroxidation and DNA damage in vitro. However, few research studies have determined the effect of coffee roasting degree on its antioxidant and anti-inflammatory effects using physiological models [23,24,25].

Previously, we have reported that the antioxidant functions and anti-inflammatory effects of coffee extracts from coffees with different levels of roasting are different in mouse hepatocytes and macrophage cell lines [11]. However, actual physiological conditions in animals have not been used to demonstrate the antioxidant and anti-inflammatory effect of coffee. Therefore, the objective of this study was to investigate the antioxidant activity and anti-inflammatory effects of coffee with different roasting levels using a mouse model in which septic shock was induced via peritoneal injection of lipopolysaccharide (LPS).

## 2. Materials and Methods 

### 2.1. Coffee Extracts

Green coffee beans (200 g, *Coffea arabica L*, Brazil Ipanema Euro Natural, Coiners International Ltd., Bucheon-si, Korea) were roasted with a Kn-8828-2 coffee roaster (Hottop USA, Cranston, RI, USA). Four different degrees of roasting were used in this experiment. Actual roasting was carried out after initial roasting. Roasting conditions including initial and final roasting temperature, and roasting time, followed those described in our previous study [23].

For coffee extraction, these roasted coffee beans were ground with a coffee grinder (900 N, Yang-Chia Machine Works, Co. Ltd., Taichung, Taiwan). Ground coffee (18 g) was then extracted via a commercial espresso machine (Faema E98; Faema, Milan, Italy). Approximately 60 mL of coffee extraction with a two-shot option was performed at 9 atmospheres in 98 °C steam pre-set by the manufacturer. The extraction was carried out three times. Extracts were stored after freeze-drying (FD5805; IlshinBioBase Co., Ltd., Dongducheon-si, Korea). The roasting conditions are summarized in Table 1.

### 2.2. Animal Experiments

Ten-week-old male C57BL/6 mice were purchased from Samtako (Osan, Korea). They were housed for one week before the start of the experiment. They were maintained at 20 ± 3 °C with 60% humidity and 12:12-h light–dark cycle. These mice were allowed free access to commercial standard mouse chow and tap water ad libitum. After one week of the adaptation period, mice were randomly divided into eight groups (*n* = 6 per group): Control, L (Light-roasted coffee extracts), F (French-roasted coffee extracts), LPS, L + LPS (Light-roasted coffee extracts with LPS), M + LPS (Medium-roasted coffee extracts with LPS), C + LPS (City-roasted coffee extracts with LPS), F + LPS (French-roasted coffee extracts with LPS). For 10 days, pretreatment groups were administrated by gavage feeding (300 mg/kg BW/day) with coffee extracts. The control and LPS groups were orally administered with DW. One hour after the last pretreatment, LPS was injected intraperitoneally at 15 mg/Kg.B.W to LPS groups. LPS was freshly dissolved in phosphate-buffered saline (PBS). Control, Light, and French groups were i.p. injected with PBS.

All experimental procedures were approved by the Institutional Animal Care and Use Committee (IACUC) at Ewha Womans University (approved number 16-068).

### 2.3. Serum Alanine Aminotransferase

ALT activities were examined using a commercial kit (Asan Pharm, Hwaseong, Korea) based on the manufacturer’s manual.

### 2.4. GSH Concentration

GSH concentration was determined using GSH reductase (Sigma-Aldrich, St. Louis, MO, USA). Liver tissues were homogenized in 10-fold PBS solution and centrifuged at 14,000 RPM for 30 min at 4 °C. The supernatant of 0.4 mL was added to 0.4 mL 0.6 M perchloric acid to precipitate tissue proteins. Glutathione concentration was then determined. Briefly, 1 mL of GSH standards (Sigma-Aldrich), sample, and 2.5 mL of reaction buffer (0.15 mM NADPH (Sigma-Aldrich), 0.1 mM 5, 5′-dithio-bis-(2-nitrobenzoic acid) (Sigma-Aldrich), 50 mM NaPO_4_ (Junsei Chemical, Chuo-ku, Tokyo), 1.5 mM etnylenediaminetetraacetic acid (E5124; Sigma-Aldrich) and 0.1 mL GSH reductase (10 units/mL)) were mixed together. Absorbance at 412 nm was measured at 0 and 60 seconds using a Biochrom Libra S50 spectrophotometer (Biochemical Ltd., Cambridge, UK). The concentration of GSH was calculated as nmol/mg protein.

### 2.5. RNA Isolation and Quantitative Real-Time Reverse Transcriptase-PCR

Total RNA was extracted from liver tissues using Trizol (Invitrogen, Carlsbad, CA, USA). RNA was transcribed into complementary DNA (cDNA) using RevertAid First Strand cDNA Synthesis Kit (Thermo Fisher Scientific, Waltham, MA, USA), which was then used as a template for a quantitative polymerase chain reaction (qPCR). qPCR was performed using Maxima SYBR Green qPCR Master Mixes (Thermo Fisher Scientific). The following primer sequences were used: glutathione synthetase (GS): 5′-GCCT GAAT CGCT CAGA TTAC-3′ (forward), 5′-CAAA GCTG GCAG AGAT AGTG-3′ (reverse), glutamate-cysteine ligase catalytic subunit (GCLC): 5′-GTGT CCGC TCTT CCAT TAC-3′ (forward), 5′-GCTC CTGC AAAC TAGA GAAG-3′ (reverse), glutamate-cysteine ligase modifier subunit (GCLM): 5′-GTGT GATG CCAC CAG ATT-3′ (forward), 5′-CTCA GAGA GCAG TTCT TTCG-3′ (reverse), glutathione peroxidase (GPx): 5′-CGAC ATTG CCTG GAA CTT-3′ (forward), 5′-GACA GCAG GGTT TCTA TGTC-3′ (reverse), tumor necrosis factor-α (TNF-α): 5′-CCTA TGTC TCAG CCTC TTCT-3′ (forward), 5′-GGGA ACTT CTCA TCCC TTTG-3′ (reverse), inducible nitric oxide synthase (iNOS): 5′-GGAA GAAA TGCA GGAG ATGG-3′ (forward), 5′-GAAC GTAG ACCT TGGG TTTG-3′ (reverse), cyclooxygenase 2 (COX-2): 5′-GTAC CGAC ATGG AGTA AACAG-3′ (forward), 5′-GACA ATCC CTGA CATG GTATC-3′ (reverse), interleukin 6 (IL-6): 5′-TCAC AGAA GGAG TGGC TAAG-3′ (forward), 5′-CACTA GGTT TGCC GAGT AGA-3′ (reverse), interleukin 1 beta (IL-1β): 5′-TCAC AAGC AGAG CAC AAG-3′ (forward), 5′-GAAA CAGT CCAG CCCA TAC-3′ (reverse), interleukin 1 alpha (IL-1α): 5′-CCTG TAAC AGAC CTCA AGA AGG-3′ (forward), 5-CCGT CAAG CTCA GAGG ATTT-3′ (reverse), and interleukin 10 (IL-10): 5′-TGAA TTCC CTGG GTG AGA-3′ (forward), 5′-CCAC TGCC TTGC TCTT ATT-3′ (reverse).

### 2.6. Luciferase Assay

RAW 264.7 mouse macrophage cells were obtained from the American Type Culture Collection (ATCC, Manassas, VA, USA). Cells were maintained in DMEM, supplemented with 10% fetal bovine serum (FBS) at 37 °C in a humidified incubator with 5% CO_2_. Cells were seeded into six-well plates at a cell density of 0.2 × 10^6^ cells/well at 4 h prior to transfection. Cells were transfected with 10–20 μg luciferase reporter DNA using Lipofectamine LTX & PLUS reagent (Invitrogen, Carlsbad, CA, USA) according to the manufacturer’s instructions. To determine the anti-inflammatory effect of coffee extracts, different roasting degrees of coffee extracts (0.5 and 1.0 mg/mL) were added to the medium (5%, *v*/*v*). After pretreatment, they were stimulated with lipopolysaccharide (LPS) for 4 h. After treatments, luciferase activity was assayed using Dual-luciferase reporter assay system (Promega, Madison, WI, USA).

### 2.7. Statistical Analysis

Results are expressed as the Mean ± SEM. All data were analyzed using the Statistical Analysis System package ver. 9.3 (SAS Institute, Inc., Cary, NC, USA). Differences between treatment groups were analyzed using one-way analysis of variance (ANOVA), followed by post hoc Duncan’s test. A *p*-value of less than 0.05 was considered statistically significant.

## 3. Results

### 3.1. Changes in Body Weight in Mice

For 10 days, C57BL/6 mice were pretreated with coffee extracts or water by gavage feeding. On the second day of the experiment, weight loss was observed in all tested groups. However, body weight was increased after the initial loss on the second day. Mean body weights of mice at the beginning and the end of treatment were 24.93 ± 0.71 g, and 25.37 ± 0.91 g, respectively. There was no significant difference in body weight among groups on the first day or the last day of pre-treatment. The total whole body weight of the mice was increased by 0.44 ± 0.22 g during the experimental period. In the same period, the mean body weight of mice of the experimental group did not show any significant difference, indicating that different roasting degrees with coffee extracts used in the experiment had no significant effect on the body weight of mice.

### 3.2. Effects of Coffee Extracts with Various Roasting Degrees on Hepatic Histopathological Changes

**Figure 1:**
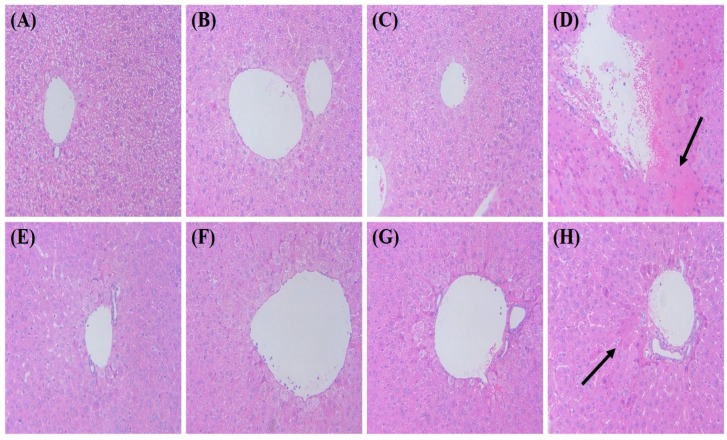
Effects of coffee extracts with various roasting degrees on histopathological changes in LPS (15 mg/Kg.B.W/day)-injected C57BL/6 mice. Ten-week-old male C57BL/6 mice were pretreated with four different roasting degrees of coffee extracts (300 mg/Kg.B.W/day) for 10 days. Liver tissues were stained with hematoxylin and eosin (Original magnification 200×). Arrows indicate liver necrosis surrounding hepatic portal vein. (**A**) Control; (**B**) Light; (**C**) French; (**D**) LPS; (**E**) Light + LPS; (**F**) Medium + LPS; (**G**) City + LPS; (**H**) French + LPS. LPS, lipopolysaccharide.

For histopathological analysis, liver tissues were stained with hematoxylin and eosin. Hepatic portal vein invasion and liver necrosis were severe in LPS treated group. These phenomena were alleviated when the mice were pretreated with Medium- or City-roasted coffee extract (Figure 1). However, when the mice were pretreated with French-roasted coffee extracts, liver necrosis and inflammatory cell infiltrations were increased.

### 3.3. Changes in Serum Alanine Aminotransferase Levels after Treatment with Coffee Extracts with Various Roasting Degrees

A serum biochemical marker of liver injury, the alanine aminotransferase (ALT) level, was measured. The serum level of ALT increased in all of the LPS-treated groups compared to the control group. Serum ALT levels decreased in groups treated with coffee extracts. The changes were about 20% in the Light + LPS and French + LPS groups (data not shown). However, the magnitude of changes in ALT level was within the reference range for ALT levels in normal mice [26]. These results showed that acute severe liver toxicity was not induced in the mice model with LPS, nor with coffee extract treatment.

### 3.4. Effects of Coffee Extracts with Various Roasting Degrees on Hepatic Glutathione Concentration

**Figure 2:**
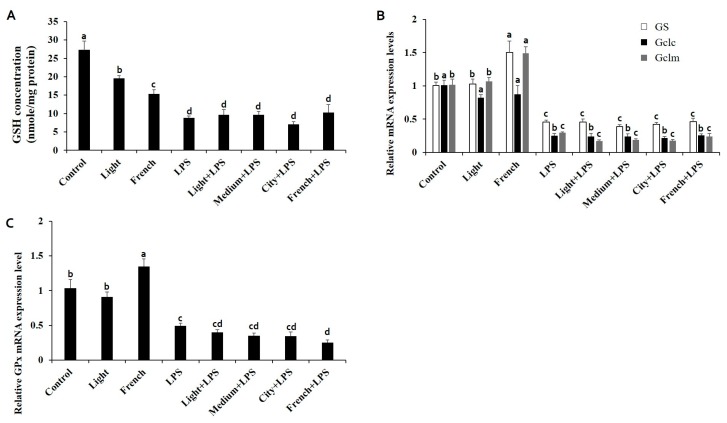
(**A**) Effects of coffee extracts with various roasting degrees on hepatic GSH concentration in LPS (15 mg/Kg.B.W/day)-injected C57BL/6 mice. Ten-week-old male C57BL/6 mice were pretreated with coffee extracts (300 mg/Kg.B.W/day) with four different roasting degrees for 10 days. mRNA expression levels of (**B**) GS, GCLC, GCLM, and (**C**) GPx were then analyzed by qRT-PCR and β-actin, housekeeping gene was used as an internal control. Different alphabets indicate significant differences (*p* < 0.05). Each bar represents the mean ± standard error (*n* = 6). GSH, glutathione; LPS, lipopolysaccharide; GS, glutathione synthase; GCLC, glutathione-cysteine ligase catalytic subunit; GCLM, glutathione-cysteine ligase modifier subunit; GPx, glutathione peroxidase; qRT-PCR, quantitative real-time reverse transcriptase-PCR.

To evaluate the antioxidant capacity of coffee extracts with a different roasting level in mice, glutathione (GSH) concentrations were measured in liver tissues (Figure 2A). The GSH concentration was decreased after LPS stimulation, but it was not ameliorated by coffee extract treatments. Hepatic GSH concentrations in coffee-only-treated groups were significantly decreased with an increasing degree of coffee roasting (Figure 2A).

### 3.5. Effects of Coffee Extracts with Various Roasting Degrees on mRNA Expression Levels of GSH Synthesizing and Peroxidase Enzymes

Glutathione synthase (GS), glutamate-cysteine ligase catalytic (GCLC), and modifier subunit (GCLM) are key enzymes of glutathione synthesis. Glutathione peroxidase (GPx) is also an enzyme that reduces hydrogen peroxide to water to remove free radicals [27,28]. In the LPS-treated group, mRNA expression levels of GS, GCLC, GCLM, and GPx were significantly decreased (Figure 2B,C). When the degree of coffee roasting was increased, the mRNA expression levels of GS, GCLC, GCLM, and GPx were increased under normal biological conditions. However, no coffee treatment affected the mRNA expression levels of GSH synthesizing enzyme or GPx under LPS stimulated conditions.

### 3.6. Effects of Coffee Extracts with Various Roasting Degrees on mRNA Expression Levels of Inflammatory Markers

**Figure 3:**
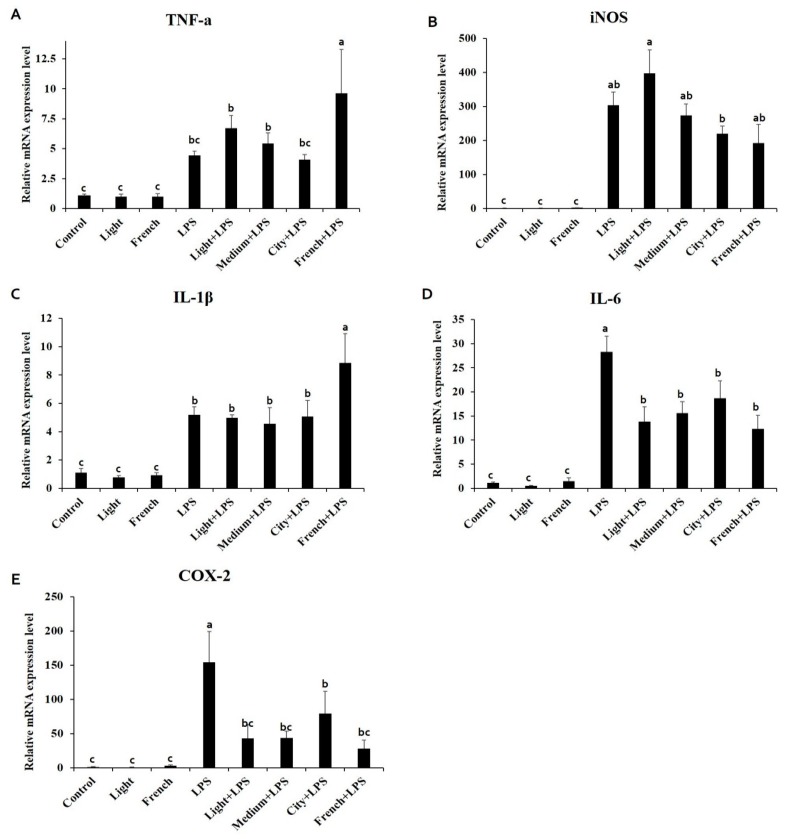
Effects of coffee extracts with different roasting degrees on mRNA expression levels of genes related to pro-inflammatory cytokines in LPS (15 mg/Kg.B.W/day)-injected C57BL/6 mice. Ten-week-old male C57BL/6 mice were pretreated with coffee extracts (300 mg/Kg.B.W/day) of four different roasting degrees for 10 days. mRNA expression levels of (**A**) TNF-α, (**B**) iNOS, (**C**) IL-1β, (**D**) IL-6, (**E**) COX-2 were analyzed by qRT-PCR and β-actin was used as a housekeeping gene. Different alphabets indicate significant differences (*p* < 0.05). Each bar represents the mean ± standard error (*n* = 6). LPS, lipopolysaccharide; TNF-α, tumor necrosis factor-alpha; iNOS, inducible nitric oxide synthase; IL-1β, interleukin 1-beta; IL-6, interleukin 6; COX-2, cyclooxygenase 2; qRT-PCR, quantitative real-time reverse transcriptase-PCR.

To investigate the anti-inflammatory effect of coffee extracts with different roasting degrees, mRNA expression levels of pro- and anti-inflammatory markers were measured by quantitative real-time PCR (qRT-PCR). Results showed that mRNA expression levels of tumor necrosis factor-alpha (TNF-α), interleukin 6 (IL-6), inducible nitric oxide synthase (iNOS), interleukin 1-beta (IL-1β), and cyclooxygenase 2 (COX-2) were increased by LPS stimulation (Figure 3). The mRNA expression levels of TNF-α and IL-1β were not changed in coffee-treated groups after LPS treatment. However, IL-6 and COX-2 levels were decreased when coffee extracts were pre-treated before LPS treatment. Nevertheless, TNF-α and IL-1β mRNA expression levels were significantly increased by French + LPS treatment. On the other hand, the iNOS mRNA expression level was decreased linearly with increasing coffee roasting degree. Upregulated mRNA expression levels of IL-6 and COX-2 by LPS treatment were significantly ameliorated with coffee extract treatments and French + LPS treatment group showed the biggest fall, although not statistically significant compared to the levels in other coffee-treated groups.

**Figure 4:**
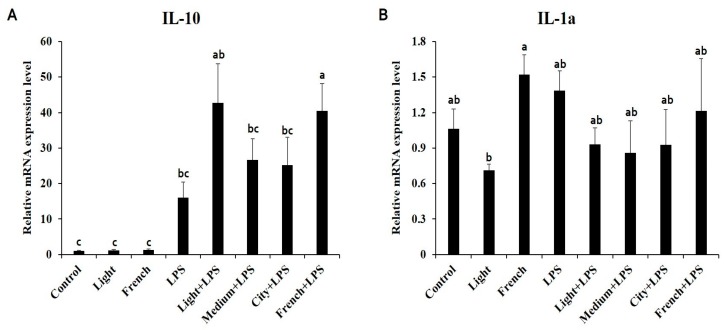
Effects of coffee extracts with different roasting degrees on mRNA expression levels of genes related to anti-inflammatory cytokines in LPS (15 mg/Kg.B.W/day)-injected C57BL/6 mice. Ten-week-old male C57BL/6 mice were pretreated with coffee extracts (300 mg/Kg.B.W/day) of four different roasting degrees for 10 days. The mRNA expression levels of (**A**) IL-10 and (**B**) IL-1α were measured by qRT-PCR and β-actin was used as a housekeeping gene. Different alphabets indicate significant differences (*p* < 0.05). Each bar represents the mean ± standard error (*n* = 6). LPS, lipopolysaccharide; IL-10, interleukin 10; IL-1α, interleukin 1-alpha; qRT-PCR, quantitative real-time reverse transcriptase-PCR.

Both interleukin 10 (IL-10) and interleukin 1-alpha (IL-1α) mRNA expression levels were increased by LPS stimulation (Figure 4). Treatment with coffee extracts tended to further increase mRNA expression of IL-10, especially the French + LPS treatment, which significantly increased IL-10 mRNA expression. However, treatment with coffee extracts did not affect IL-1α mRNA expression.

### 3.7. Effects of Coffee Extracts with Various Roasting Degrees on Nf-κB and ARE Activation

**Figure 5:**
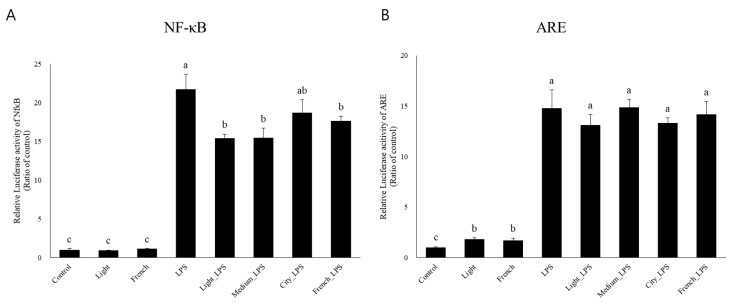
Effects of coffee extracts with different roasting degrees on promoter activity levels of NF-κB and ARE in LPS-stimulated RAW 264.7 cells. Cells were transfected with (**A**) NF-κB or (**B**) ARE reporter and co-transfected with pR-K-Renilla for internal normalization. At 24 h after transfection, cells were pretreated with four different roasting degrees of coffee extracts for 24 h and LPS was added. After four hours of LPS stimulation, total cells were lysed and luciferase activities were measured with a dual-luciferase system. Different alphabets indicate significant differences (*p* < 0.05). Each bar represents the mean ± standard error (*n* = 3). LPS, lipopolysaccharide; NF- κB, nuclear factor kappa-light-chain-enhancer of activated B cells; ARE, antioxidant response element.

To investigate the mechanism involved in the anti-inflammatory effect of coffee extracts with different roasting degrees, luciferase reporter assays were conducted to measure transcriptional activation of NF-κB and antioxidant response element (ARE). LPS treatment initiated Nf-κB activation in RAW 264.7 cells (Figure 5A). Coffee extract significantly decreased the transcriptional activation of Nf-κB in LPS-stimulated status regardless of the roasting degree. Coffee extracts increased ARE luciferase activity (Figure 5B). In addition, LPS increased the reporter gene activity of ARE. However, coffee extracts did not affect ARE activation when cells were stimulated by LPS.

## 4. Discussion and Conclusions

Previous studies have demonstrated that coffee extracts possess antioxidant or anti-inflammatory effects both in vitro and in vivo. However, to the best of our knowledge, no studies have reported the antioxidant and anti-inflammatory effects of coffee extracts with different roasting degrees using an actual live animal model. Hence, the aim of the present study was to examine the effects of coffee extracts with different roasting degrees on the antioxidant and anti-inflammatory system using a mouse model in which septic shock was induced via peritoneal injection of LPS.

By referring to previous studies [29,30], each mouse received 300 mg of coffee powder per kg body weight in this study. This corresponds to 10 L of ready-made coffee or about 0.7 kg of coffee beans consumed by a 70-kg person. Since mice have a metabolic rate 7–10 times higher than humans’ [25,31], this dose corresponds to 1 to 1.4 L of coffee a day in a person with an average body weight.

Several studies have reported that the antioxidant capacity of the coffee extracts is reduced as coffee roasting degree increases based on chemical assessment [11,13,14,21]. Since the antioxidant functions of roasted coffee extracts should affect physiological metabolism in animals, it is important to assess the biological antioxidant capacity of coffee extracts with different degrees of roasting using animal models. GSH is one of the major indicators of oxidative stress. To assess the oxidative stress and antioxidant effect of coffee extracts with different roasting degrees, we measured GSH concentration using 10-week-old male C57BL/6 mice treated with LPS. Hepatic intracellular GSH concentration was reduced by LPS stimulation, but the decrease was not ameliorated by coffee extract treatments (Figure 2A). Hepatic intracellular GSH concentration was the highest in the group treated with extract from Light-roasted coffee. With the increasing roasting degree of coffee, hepatic intracellular GSH concentration decreased more in non-LPS treated groups. To confirm the cellular mechanism of intracellular GSH reduction by roasting, genes related to GSH synthesis were analyzed. GS is an important enzyme for the synthesis of GSH. GCLC and GCLM are rate-limiting enzymes of GSH synthesis. GPx is also an enzyme that reduces free radicals by oxidizing GSH to convert hydrogen peroxide into water [27,28]. Although coffee extracts did not significantly affect gene expression under LPS-stimulated conditions, gene expression levels of GS, GCLC, GCLM, and GPx were significantly increased by French-roasted coffee extracts under physiological conditions without LPS stimulation (Figure 2B,C). These results indicate that coffee roasting level does not dramatically affect the physiological antioxidant system in LPS-challenged mice, contrary to the results of previous mouse hepatocyte cellular experiments [11].

Caffeine and chlorogenic acids (CGAs), as major constituents of coffee, (in)directly play an important role in the antioxidant capacity of coffee extracts [21,22]. It is well known that the roasting process affects chemical constituents of coffee extracts due to high temperatures [32,33]. Previous studies have reported that the proportion of caffeine is constant even when the degree of coffee roasting is increased. However, phenolic compounds including CGAs are significantly decreased, while Maillard Reaction Products (MRPs) are increased during roasting [10,11]. These observations suggest that CGAs can be integrated into melanoidins as new products produced by the Maillard reaction during roasting [34,35,36]. The results of the present study in Figure 2 also indicated that coffee extracts significantly upregulated GS, GCLC, GCLM, and GPx mRNA expression levels if the roasting degree was above a certain level when LPS was not treated. These results indicated that MRPs might have been formed, although the GSH concentration was gradually reduced as the coffee roasting degree was increased (Figure 2).

In both animals and humans, oxidative stress is a major cause of inflammation. Various inflammatory mediators including NO, pro-inflammatory cytokines, and adhesion molecules are closely related to inflammatory symptoms such as pain, fever, redness, swelling, and loss of cell function during inflammatory responses [37]. Therefore, it is important to modulate the inflammatory response appropriately. Pro-inflammatory cytokines such as TNF-α, IL-6, and IL-1β are increased by oxidative stress and inflammatory responses. NO is also produced from L-arginine amino acid by the enzymatic action of iNOS, activated during an inflammatory reaction [37]. In addition, COX-2, induced by a variety of inflammatory mediators, is an important factor for prostaglandin production in the inflammation process [38]. In this study, mRNA expression levels of TNF-α and IL-1β surged by LPS stimulation were significantly increased by additional French coffee extract (Figure 3A,C). An epidemiological study has reported that coffee consumption increases TNF-α concentration in serum [39]. However, many previous studies have reported that coffee extracts reduce TNF-α and IL-6 mRNA expression levels both in vitro and in vivo [11,15,40]. On the other hand, mRNA expression levels of iNOS, IL-6, and COX-2 decreased as the roasting degree of coffee increased (Figure 3B,D,E). The mRNA expression of iNOS was decreased significantly in city + LPS; however, other coffee-treated groups with LPS activation also tended to decrease in iNOS mRNA expression level. Interleukin 10 (IL-10) is an anti-inflammatory cytokine that predominantly inhibits LPS mediated induction of pro-inflammatory cytokines TNF-α, IL-1β, and IL-12 secretion, which blocks NF-κB activation [41,42]. Mice treated with LPS treatment showed increased IL-10 expression in this study (Figure 4A). Treatments with coffee extracts in LPS-challenged mice increased IL-10 expression compared to LPS treatment alone. This indicates that coffee extracts might have anti-inflammatory effects through regulating the NF-κB signaling pathway. In order to determine whether or not coffee extracts could regulate inflammatory responses via NF-κB activation, we conducted a luciferase reporter assay of NF-κB in LPS-stimulated RAW 264.7 cells and evaluated the regulatory effect of coffee extracts. Our results demonstrated that coffee extracts decreased NF-κB activation when cells were stimulated with LPS (Figure 5A). It has been reported that caffeine can decrease LPS-induced TNF-α, IL-6, and iNOS secretion by modulating NF-κB activation [43]. CGAs, as major components of coffee extracts, can also downregulate NF-κB signaling, thereby lowering the secretion of iNOS, IL-6, and COX-2 [44,45,46]. In our previous study, we have shown that coffee extracts with various roasting degrees possess antioxidant properties that might be responsible for its anti-inflammatory effects. In order to determine the antioxidant protective mechanism exerted by coffee extracts in an LPS-stimulated state, we investigated the effect of coffee extracts on the activation of ARE in mice. We observed that coffee-extract-only treatments (both Light & French) significantly increased ARE activation compared to the control, which could induce the genes related to the cellular antioxidant system. When mice were stimulated with LPS, ARE activation was increased significantly more compared to in the control group and coffee-only-treated groups. LPS is known to trigger ARE activation to protect against inflammatory oxidative stress. However, coffee extracts had no additional effect on ARE transcriptional activity if there was LPS stimulation.

Our results indicated that the degree of inflammatory cytokine mRNA expression was affected by coffee roasting in the LPS-treated mice model. This did not conform to our previous results using an in vitro model [11]. The complexity of the physiological system in the animal model might have resulted in such differences between cell models and animal models. Anti-inflammatory effects of coffee extracts were found to be different depending on the roasting degree. This might be due to the fact that the proportion of constituents in coffee extracts such as caffeine and CGAs is changed by the roasting process [47].

## Figures and Tables

**Table 1:** Initial weight, initial temperature, final temperature, roasting time, roasting weight, percentage of loss, and roasting degree.

Sample	Initial Weight (g)	Initial Temperature	Final Temperature	Roasting Time (min)	Final Weight (g)	Loss (%)	Roasting Degree	
							Degree	Agtron No.
R1	200	180 °C	199 °C	8.00	177.0	11.5	Light	94.0 ± 1.4
R2	200	180 °C	204 °C	9.00	172.6	13.7	Medium	75.9 ± 1.9
R3	200	180 °C	209 °C	10.33	168.2	15.9	City	57.7 ± 1.6
R4	200	180 °C	212 °C	11.33	153.6	23.2	French	32.2 ± 0.3
